# Exploring Community Smokers’ Perspectives for Developing a Chat-Based Smoking Cessation Intervention Delivered Through Mobile Instant Messaging: Qualitative Study

**DOI:** 10.2196/11954

**Published:** 2019-01-31

**Authors:** Tzu Tsun Luk, Sze Wing Wong, Jung Jae Lee, Sophia Siu-Chee Chan, Tai Hing Lam, Man Ping Wang

**Affiliations:** 1 School of Nursing, The University of Hong Kong Hong Kong China (Hong Kong); 2 School of Public Health, The University of Hong Kong Hong Kong China (Hong Kong)

**Keywords:** chat intervention, instant messaging, mHealth, mobile phone, social media, smoking cessation, tobacco dependence, WhatsApp

## Abstract

**Background:**

Advances in mobile communication technologies provide a promising avenue for the delivery of tobacco dependence treatment. Although mobile instant messaging (IM) apps (eg, WhatsApp, Facebook messenger, and WeChat) are an inexpensive and widely used communication tool, evidence on its use for promoting health behavior, including smoking cessation, is scarce.

**Objective:**

This study aims to explore the perception of using mobile IM as a modality to deliver a proposed chat intervention for smoking cessation in community smokers in Hong Kong, where the proportion of smartphone use is among the highest in the world.

**Methods:**

We conducted 5 focus group, semistructured qualitative interviews on a purposive sample of 15 male and 6 female current cigarette smokers (age 23-68 years) recruited from the community in Hong Kong. All interviews were audiotaped and transcribed. Two investigators independently analyzed the transcripts using thematic analyses.

**Results:**

Participants considered mobile IM as a feasible and acceptable platform for the delivery of a supportive smoking cessation intervention. The ability to provide more personalized and adaptive behavioral support was regarded as the most valued utility of the IM–based intervention. Other perceived utilities included improved perceived psychosocial support and identification of motivator to quit. In addition, participants provided suggestions on the content and design of the intervention, which may improve the acceptability and usability of the IM–based intervention. These include avoiding health warning information, positive messaging, using former smokers as counselors, and adjusting the language style (spoken vs written) according to the recipients’ preference.

**Conclusions:**

This qualitative study provides the first evidence that mobile IM may be an alternative mobile health platform for the delivery of a smoking cessation intervention. Furthermore, the findings inform the development of a chat-based, IM smoking cessation program being evaluated in a community trial.

## Introduction

Burgeoning mobile communication technologies have provided a promising means to deliver mobile health (mHealth) intervention, including behavioral change treatment [1,2]. The widespread use of mobile devices has rendered mobile smoking cessation program a scalable measure to combat tobacco use, the leading modifiable cause of morbidity and mortality worldwide [3]. Commonly studied mHealth interventions for treating tobacco use include short message service (SMS) text messaging support [4], social media (eg, Facebook and Twitter) [5], and smartphone apps [6]. Substantial research has found that SMS text messaging-based interventions are effective in promoting quitting, though the evidence base for other mHealth cessation modalities is still developing [4,7]. Some SMS text messaging programs personalized the intervention contents according to the smokers’ characteristics and allowed 2-way communication wherein a recipient can text keywords, such as “crave,” to receive on-demand support [8,9]. However, it remains unknown if chat messaging interventions, which feature more personalized and 2-way communication between smokers and treatment providers, may further improve smoking cessation outcomes.

Mobile instant messaging (IM) apps (eg, WhatsApp and WeChat), which allow the exchange of text, emojis, voice messages, and multimedia files freely through the internet, have rapidly replaced SMS as the most widely used mobile communication tool [10]. We found that health information exposure from IM was associated with healthier behaviors, including more frequent physical activity and less smoking, suggesting IM may be an alternative and potentially effective mHealth tool for delivering behavioral interventions [11]. Despite a growing interest in integrating IM into health care, emerging research has largely focused on using IM to promote clinical patient management and interprofessional communication [12-14]. The effectiveness of using IM for promoting smoking cessation and other health behaviors remains largely untested. Recent reviews identified only 2 related studies in the literature [12,14]. The first study is a randomized controlled trial we piloted, which found that an intervention using WhatsApp social group was effective in preventing smoking relapse in recent quitters [15]. Another study evaluated a WhatsApp-based physical activity program, which showed small beneficial effects on physical fitness and cardiovascular risk [16].

Hong Kong is the most developed and westernized city of China with stringent tobacco control measures. The daily cigarette smoking rate of 10.0% in 2017 is among the lowest in the world [17]. Novel interventions that motivate and assist the remaining smokers to quit can be one of the solutions to reduce the smoking rate to 5% for implementing tobacco endgame policies [18]. Leveraging the extensive smartphone penetration in the local population (88.6% in 2017) [17], we proposed to develop a chat-based smoking cessation intervention delivered through mobile IM to be evaluated in a community-based trial. Given the lack of similar prior research, we conducted a formative qualitative study to inform the content and design of the intervention in our target population. This study aims to explore the perception of the proposed mobile IM intervention for smoking cessation in community Chinese smokers.

## Methods

### The Proposed Intervention

The proposed mobile IM intervention for smoking cessation consists of 2 major components. The first component is a chat-based intervention wherein a smoker can chat individually (one-to-one) with a trained counselor through IM, who will provide real-time behavioral support to help the smoker achieve abstinence. The second component consists of messages on information about smoking cessation regularly sent by the counselor through IM. This study determined the contents of both intervention components.

### Study Design and Participants

We conducted this qualitative study in a purposive sample of Chinese community smokers in Hong Kong using semistructured, focus group interviews. The eligibility criteria include the following: (1) being a Hong Kong resident aged ≥18 years; (2) ability to communicate in Cantonese; (3) currently smoking cigarettes (at least weekly); and (4) having used IM apps installed on a smartphone. Subjects who were physically or mentally unable to communicate were excluded. To increase the heterogeneity of the sample, we purposefully selected subjects of different sex, age group, and smoking pattern (daily/nondaily). The Public Opinion Programme (POP) of the University of Hong Kong, one of the leading local survey agencies, was commissioned to recruit subjects by telephone contact to potentially eligible households. Persons who answered the phone were asked if there was a residing household member that met the purposive sampling criteria. If so, the member was then invited to participate in the focus group interview. Subject recruitment ceased after data saturation.

This study was approved by the Institutional Review Board of the University of Hong Kong/Hospital Authority Hong Kong West Cluster (UW 17-206). We obtained informed written consent from all participants before the interview began.

### Study Procedures

All interviews were conducted in the evening in a quiet meeting room arranged by the POP at the University of Hong Kong. Before the interviews began, participants completed a one-page questionnaire on their sociodemographic characteristics and pattern of cigarette smoking.

A smoking cessation research nurse and an experienced counselor conducted the focus group interviews. No other personnel apart from participants and researchers were present throughout the interviews. The focus group discussion began with the interviewer asking participants to describe their smoking patterns and previous quit attempt as opening questions. Then, following an interview guide, the interviewer introduced the 2 components of the proposed mobile IM intervention for smoking cessation and asked open-ended interview questions (eg, what is your view on using mobile instant messaging for smoking cessation? What is your suggestion on the intervention content to strengthen your motivation to quit?). Probes (eg, “tell me more…”) were used where appropriate to illicit more in-depth responses. All interviews were conducted in Cantonese and audiorecorded with participants’ consent. Each participant was reimbursed HK $150 (HK $7.8 ≈ US $1) in cash for their time and travel expenses.

### Data Analyses

The audio records were transcribed verbatim. Interim analyses were performed following each focus group to refine the interview questions. Two investigators independently analyzed the transcripts following the principles of thematic analyses, as described by Braun and Clarke [19]. Researchers first familiarized themselves with the data by reading all the transcripts line-by-line to generate initial thoughts on the data. Next, all passages pertaining to the research question were coded. Codes that shared similar meaning were then clustered into subcategories. Broader themes were then searched, reviewed, and defined by collating conceptually similar subcategories. Discrepancies in the coding decision were resolved by reanalysis of the transcript and discussion with an additional investigator, who verified the consistency and coherence in thematic codes [20]. All analyses were performed in the original Cantonese. Selected interview excerpts were translated into English for reporting and back-translated for checking by the researchers who were bilingual (Cantonese and English).

## Results

### Sample Characteristics

We interviewed 21 smokers in 5 focus groups (about 4 participants each) from May to June 2017. Each interview lasted about 1 hour. The mean (SD, range) age of participants was 48.2 (17.1, 23-68) years and 71.4% (15/21) were male (Table 1). More than half (14/21, 67%) were daily smokers.

Thematic analyses identified 2 major themes from the data. These included (1) the perceived utility of IM for smoking cessation and (2) recommendation about the content and delivery of the proposed intervention.

### Perceived Utility of Instant Messaging for Smoking Cessation

Participants found using mobile IM for smoking cessation novel and considered IM as a feasible and acceptable platform for the delivery of cessation advice and support to smokers. Some participants preferred receiving counseling support from IM to the phone as IM is neither restricted by time nor smokers’ availability.

WhatsApp is better than telephone, you can choose when to reply and not being disturbed by telephone calls

#15, male, 38 years

Under the theme of perceived utility, we identified 4 subthemes on strategies or techniques for promoting quitting using the IM intervention as discussed by participants: (1) providing personalized behavioral support; (2) providing psychosocial support; (3) identifying motivators to quit; and (4) serving as an information center.

#### Providing Personalized Behavioral Support

Participants felt that IM could facilitate 2-way communication by which counselors could learn about the characteristics of smokers and provide more personally meaningful feedback to support their quitting. The personalized intervention could be based on age, sex, and other characteristics of smokers such as values and emotional needs.

It is very important to discuss with the smokers in a personalized context, like according to the smokers’ family background and working environment…and provide tailored response.

#7, male, 40s

You can provide more specific feedback based on their characteristic like sex. For example, females may care more about their appearance…and you can tell how smoking would affect their skin or teeth… I would be attracted to such information.

#19, female, 29 years

All of us have different emotional needs at different age, which may be related to study, work or family…if the messages can target and soothe my distress, then I may be able to skip that cigarette.

#12, female, 58 years

The importance of providing personally relevant information, which smokers could relate to, was further highlighted by participants.

Some people do not have a child…sending messages related to family and that sort of thing to them would be meaningless.

#7, male, 40s

I am already in my sixties…I wouldn’t care much about my look…so information like how smoking affects the skin would not work on me…

#20, male, 66 years

Many participants reported habitual smoking in their daily living such as taking a cigarette while commuting to work or immediately after a meal. They conceived that a counselor can appreciate their daily smoking routine and then provide more timely IM support to help them break their smoking habit.

The counselor can understand your pattern of smoking and then schedule the message accordingly.

#14, female, 53 years

You can text me at 1:30 pm…right after my lunch…ask about my progress and encourage me not to smoke that cigarette…

#11, female, 66

For those who preferred to quit progressively, participants considered IM useful for a counselor to guide their quitting through an iterative process of goal setting and follow-up. The counselor can help smokers monitor their cigarette consumption and keep track of their target.

You can set a target, like first cutting down my daily intake from 1 pack to 5 cigarettes, and then check my progress in WhatsApp. Then, you can set another target…

#19, female, 29 years

**Table 1:** Sample characteristics (N=21).

Characteristics		Participants, n (%)
Mean age (SD), years		48.2 (17.1)
Male		15 (71)
**Marital status**		
	Unmarried	8 (38)
	Married	10 (48)
	Others	3 (14)
**Education level**		
	Lower secondary (US grade 7-9)	1 (5)
	Upper secondary (US grade 10-12)	9 (43)
	Tertiary	11 (52)
**Employment status**		
	Employed	11 (52)
	Unemployed	2 (10)
	Retired or students or housekeepers	8 (38)
**Monthly personal income (in HK $; HK $7.8 ≈ US $1)**		
	≤$9999	6 (30)
	$10,000-$19,999	8 (40)
	≥$20,000	6 (30)
Having a child		10 (48)
Daily smoking (vs nondaily smoking)		14 (67)
**Number of cigarettes per day**		
	≤10	14 (67)
	11-20	6 (29)
	>21	1 (5)
**Previous quit attempts**		
	0	8 (40)
	1	5 (25)
	>2	7 (35)
**Intention to quit**		
	Planning to quit within 1 month	0 (0)
	Planning to quit between 1 and 6 months	5 (24)
	Not planning to quit in 6 months	16 (76)
**Number of instant messages sent and received per day**		
	1-20	7 (35)
	21-50	6 (30)
	51-100	4 (20)
	>100	3 (15)

#### Providing Psychosocial Support

Regular receipt of messages from a counselor via IM was considered by participants as a continuing source of psychosocial support for their quit attempts. They indicated that simple messages, like greeting or reminders that they were quitting, are helpful in creating a sense of being backed by someone who cares about their quitting and encourage them to continue through the quitting process.

I am the kind of person who needs support from others to quit smoking…I relapsed in my last quit attempt because no one acknowledged my efforts…if somebody can keep reminding and motivating me to quit through those messages, I think it would work on me.

#9, male, 42 years

I want to quit but have found it very difficult as all of my colleagues smokes…having someone encouraging and following up on my progress can push me harder to stop smoking

#19, female, 29 years

In addition, participants suggested that the intervention can empower their significant others, like their family members, through IM to provide additional, potentially stronger psychosocial support for their quitting.

You might as well approach my son through WhatsApp and ask him to relay the messages to me…it would be warmer to hear face-to-face from my son saying “Daddy, will you please smoke less?”…

#15, male, 38 years

#### Identifying Motivators to Quit

Participants emphasized the importance of having a reason to quit or reduce their cigarette consumption based on their personal values, which may be related to their health, image (as smoking is severely denormalized in Hong Kong), and family. They remarked a counselor can help them identify their values (often described as a “weakness point”) through IM, which can be exploited to strengthen their commitment to become smoke-free.

I think personalized messaging is paramount to finding a reason that can prompt the smoker to stop smoking.

#4, male, 29 years

I believe everyone has a trigger point for quitting, and through chatting in WhatsApp you can find the trigger point and motivate the smokers to quit.

#19, female, 29 years

Through conversation you will be able to identify and “prick” the smoker’s weakness point to stop smoking…we all have one.

#7, male, 40s

#### Serving as an Information Center

Some participants noted that IM could act as an accessible platform where they can obtain knowledge about methods of quitting (eg, how to use smoking cessation medications) and inquire about other information related to tobacco use.

WhatsApp can provide a channel for smokers to ask about methods to quit…like how to quit progressively, or how to use the nicotine gum and patch.

#20, male, 66 years

There are tobacco products other than cigarettes like electronic cigarettes or IQOS…WhatsApp allows the smokers to obtain more information about them…

#17, male, 23 years

Furthermore, they thought they could refer back to the previous conversation with the counselor for information about quitting in times of need such as during episodes of craving.

### Design Consideration of the Intervention

Participants provided suggestions on the content of the intervention and methods of delivery to optimize the proposed IM smoking cessation support program. These suggestions fell into 4 broad categories as follows: (1) avoid health warnings; (2) positive messaging; (3) former smokers as counselors; and (4) text language.

#### Avoid Health Warnings

When asked about health information to be included in the proposed intervention, participants overwhelmingly disapproved messages related to the negative consequence of smoking. They referred to such information as unnecessary, unhelpful, and aversive.

I am aware of the risk of smoking posed, like those labelled on the cigarette packages, and it doesn’t work on me…I would feel annoyed to be reminded of these warnings [3, male, 25].

I believe 9.9 out of 10 smokers know the harms of smoking; all of us can list them…You can provide such information only when we ask for it.

#8, female, 25

In addition, participants suggested health warning information may be counterproductive and may trigger their desire to smoke as a gesture of defiance.

I would feel disgusted if someone keep telling me about the adverse effect of smoking…and I might be prompted to smoke…like saying “it’s none of your business.”

#9, male, 42

#### Positive Messaging

Following their criticism on health warning information, participants suggested a counselor should motivate smokers using more positive, nonjudgmental messages, like reframing the harm of smoking as a benefit of quitting.

Warning is an outdated way to motivate smokers to quit…those who dread the risk of smoking would have mostly quitted already. I think a gentler approach like reward and encouragement would be better.

#15, male, 38

I think information like how smoking would disrupt your family well-being or lead to early death is rather negative…you can present them in an opposite manner, like telling how quitting could promote happiness or family harmony…instilling them hope.

#21, male, 53

In addition, participants welcomed messages about the immediate, positive impact of quitting, such as recovery of health or amount of money saved from not buying cigarettes. They believe these messages could validate and reinforce their commitment to quit.

Messages about what would happen to my body, like how my lung function would improve minutes, hours and days after I stop smoking cigarette, would encourage me to keep it up…as I wouldn’t want to ruin my progress…

#4, male, 25

#### Former Smokers as Counselors

To some participants, having a former smoker as a counselor to coach their quitting is considered helpful in strengthening the intervention. They suggested the quit advice coming from a person who successfully quit is more convincing. In addition, they believed that former smokers could better empathize with the smokers’ history of smoking and the challenge of making a quit attempt.

It is like we are in the same boat…The counsellor can relate to my feeling better.

#7, male, 40s

Smokers and nonsmokers are different…it would be easier to communicate with a counsellor who smoked before, as he or she can understand my struggles and thoughts…

#12, female, 58

#### Text Language

Responses varied among participants about the style of text language (spoken language vs written language), which appeared to be governed by their personal preference on the closeness of their relationships with a counselor. Some participants recommended messages in spoken language, describing them as more casual and amiable for a closer relationship with the counselor.

To me is spoken language…you can tell from the tone the emotion and attitude of the person you are talking with. Written language feels less lively.

#7, male, 40s

Spoken language makes you feel like chatting with a friend.

#20, male, 66

Other participants suggested that the use of written language to be more appropriate for maintaining a more formal relationship with the counselor and considered health information in written language more authentic.

written language is better for a more distanced relation to the counselor, which seems more professional…

#8, female, 25

I prefer written language…health advice in formal tone sounds more credible.

#4, male, 25

## Discussion

### Principal Findings

This qualitative study explored the perception of using mobile IM for promoting smoking cessation in community cigarette smokers in Hong Kong. Participants considered IM as a feasible and acceptable modality of delivering smoking cessation support to smokers. They brainstormed how IM could facilitate their quitting, including individual behavioral change techniques (BCTs), which promotes their motivation to quit (eg, identify reasons for wanting to stop smoking), self-control ability (goal setting), use of adjuvant cessation aids (advise on stop-smoking medication), and other supportive BCTs that features interaction (elicit and answer questions about smoking cessation) [21]. Furthermore, they provided suggestions on the content and design of the intervention, which may improve its acceptability and usability.

The ability to provide more personalized behavioral support based on the individual characteristics of smokers was considered by participants as the primary merit of the proposed chat intervention for smoking cessation using IM. A formative study for developing mHealth programs for smoking cessation in the United States also reported similar findings [22]. Crucially, personalization requires personal information, which is often difficult to gather during brief clinical interaction at the baseline owing to time constraint. Through IM, a counselor can continuously learn about the characteristic of smokers, monitor their progress, and tailor the type and intensity of behavioral change intervention that targets different aspects of the quitting process. In addition, IM may facilitate behavioral phenotyping such that the counselor can determine how smokers respond to failure (eg, slip or relapse during quitting) and then apply appropriate behavioral techniques to address their reactions [23].

Practical cessation support apart, the participants valued receiving external emotional support or a feeling of “being cared for” during their quit attempt through IM. Moreover, mechanistic evaluations of effective automated SMS text messaging programs for smoking cessation found smokers’ perceived psychosocial support as a key mediator for achieving abstinence, even when they acknowledged that messages were coming from a computer rather than a real person [24,25]. Through true person-to-person interaction and delivery of more personalized and tailored cessation support using IM, our proposed chat intervention may potentially improve their perception of psychosocial support and, thus, cessation outcomes relative to other existing SMS text messaging-based interventions, which deliver more static computer-generated responses to end users [26].

Participants suggested that the IM intervention could help clarify their personal value or “weakness point,” which was regarded as crucial motivators to quit smoking; this provides a case for adopting value-based counseling models such as motivational interviewing (MI) and acceptance and commitment therapy (ACT) [27,28]. In the context of smoking cessation, MI aims to help smokers clarify and deal with their indecision to making a quit attempt, whereas ACT aims to maximize their psychological flexibility against the negative experiences associated with smoking cessation. Both MI and ACT attempt to strengthen the smokers’ commitment to quit based on their values. Preliminary results from emerging research have suggested that ACT delivered through a smartphone platform is feasible and promising to promote quitting [29,30]. Taken together, the ACT may be a useful behavioral change model that can be incorporated into and enhance the IM intervention.

This formative study conducted in our target end users has informed the content and design of our proposed intervention. The chat intervention now adopts the counseling model of the ACT and includes all BCTs suggested by participants, which will be personalized and tailored to the need indicated by smokers. As participants showed personal preference on the text language style, the counselor would also adjust accordingly to facilitate a therapeutic relation with smokers [31]. Based on the participants’ comments on health warning information and positive messages, we also modified regular messages that were used in our previous smoking cessation trials [32,33]. Specifically, we removed loss-framed messages (eg, harm of smoking) and added gain-framed messages (eg, the short-term benefit of quitting on health) and messages that encourage smokers to quit/maintain abstinence for their values. Currently, the intervention is being evaluated in an ongoing randomized controlled trial for Chinese community smokers in Hong Kong (NCT03182790).

Compared with smartphone apps for smoking cessation, IM may be more scalable and market-ready, as many mobile IM apps are freely available and widely used. However, our proposed IM program, which requires a counselor to deliver the chat messaging intervention to recipients, may incur greater operation cost than other SMS text messaging interventions, which can be delivered through an automatic computer response system. Nevertheless, as artificial intelligence and related techniques, such as natural language processing, continue to mature, chatbots can be developed to simulate the conversation made by a human, effectively reducing the manpower and cost of chat-based intervention for smoking cessation [34].

Although this study focused on using IM for smoking cessation, it may be relevant to similar intervention for treating other health risk behaviors, such as alcohol use disorder and physical inactivity, and other interactive digital health intervention, including Chatbot. Intervention studies that implement our proposed chat-based IM intervention can generate real-world text data (dialogues between smokers and counselors), which are necessary for training the chatbots to recognize the need of smokers.

### Limitations

This study has several limitations. First, as it focused on the use of mobile IM for smoking cessation, nonsmartphone owners were excluded. The views expressed during the interview were specific to current cigarette smokers who owned a smartphone, who are typically younger, and have a higher socioeconomic position than the general population [35]. Second, as participants were recruited from the community in Hong Kong, our findings may not be applicable to clinical and non-Chinese populations. Third, the qualitative study was relatively small (N=21), although the study endpoint was determined by data saturation, which became apparent during the fourth focus group and confirmed in the fifth focus group. Finally, owing to the lack of funding, member checking (actively involving participants in verifying the results) was not conducted. Nevertheless, 2 investigators independently coded and analyzed the transcripts to improve the trustworthiness of the findings.

### Conclusions

This formative qualitative study in Chinese community smokers provides a case for developing and evaluating smoking cessation interventions delivered through IM, which may be an alternative mHealth modality for treating tobacco dependence. The findings inform the development of a chat-based smoking cessation intervention delivered through mobile IM, which is being evaluated in an ongoing randomized controlled trial for Chinese community smokers in Hong Kong.

## Abbreviations

ACT: acceptance and commitment therapy
BCT: behavioral change techniques
IM: instant messaging
mHealth: mobile health
MI: motivational interviewing
POP: Public Opinion Programme
SMS: short message service
US: upper secondary
